# Speaker-independent dysarthria severity classification using self-supervised transformers and multi-task learning

**DOI:** 10.1371/journal.pdig.0001076

**Published:** 2025-11-12

**Authors:** Balasundaram Kadirvelu, Lauren Stumpf, Sigourney Waibel, A. Aldo Faisal

**Affiliations:** 1 Brain & Behaviour Lab, Department of Computing and Department of Bioengineering, Imperial College London, London, United Kingdom; 2 Chair in Digital Health & Data Science, Faculty of Life Sciences, University of Bayreuth, Bayreuth, Germany; Liverpool John Moores University - City Campus: Liverpool John Moores University, UNITED KINGDOM OF GREAT BRITAIN AND NORTHERN IRELAND

## Abstract

Dysarthria, characterised by slurred speech, is a hallmark of many neurological disorders and brain trauma. Clinical assessment requires an audio-visual investigation by a trained healthcare expert, who evaluates criteria such as respiration, phonation, articulation, resonance, and prosody during speech. Quantitative assessment of dysarthria is challenging due to its complexity, variability, and the subjective nature of human-observation-based scoring methods. We present a novel machine-learning framework using transformers for stratifying and monitoring patient speech. Our framework integrates a wav2vec 2.0 model, pre-trained on raw speech data from healthy individuals. To reduce reliance on speaker-specific characteristics and effectively manage the intrinsic intra-class variability of dysarthric speech, we employ a contrastive learning strategy with a multi-task objective: cross-entropy loss for classifying dysarthria severity, and triplet margin loss to ensure latent embeddings are grouped by severity rather than by speaker. This Speaker-Agnostic Latent Regularisation (SALR) framework provides an objective, accessible, and cost-effective alternative to traditional assessments. On the UA-Speech dataset, SALR achieved 70.5% accuracy and 59.2% F1 using leave-one-subject-out cross-validation—a 16.5% absolute (30% relative) improvement over prior benchmarks. Explainability analysis indicates that our multi-task objective enhances the ordinal structure of the latent space, reducing dependence on speaker-specific cues and demonstrating robustness and generalisability. In conclusion, this proof-of-concept study demonstrates the potential of the SALR framework for speaker-independent dysarthria severity classification, with potential implications for broader clinical applications in automated dysarthria assessments.

## Author summary

Dysarthria, a speech impairment caused by neurological conditions, is a common symptom of a range of neurological disorders, including stroke, head trauma, brain tumours, Parkinson’s disease, multiple sclerosis, motor neuron disease, and cerebral palsy. Accurate assessment of dysarthria is challenging due to the complex nature of speech disorders, the variability among patients, and the biases inherent in human observation. Traditional methods for evaluating dysarthria are often subjective and rely heavily on expert opinions. There is a clear need for more standardised, efficient and accessible tools to assess dysarthria. We have developed a novel deep-learning framework to classify dysarthria severity levels directly from speech recordings without needing expert input. Our framework, tested using the Universal Access Speech dataset, achieved a classification accuracy of 70.5%, surpassing the previous benchmark by a 16.5% increase in accuracy. The results indicate that our framework provides a more consistent and objective way to classify dysarthria severity compared to traditional assessments. This advancement could lead to more reliable dysarthria evaluations in clinical environments, potentially impacting treatment approaches and improving patient care.

## Introduction

Dysarthria, characterised by impaired control over speech muscles due to neurological conditions, has a profound impact on communication and quality of life [1]. Dysarthria occurs in a range of neurological disorders, including stroke, Parkinson’s disease, multiple sclerosis, motor neuron disease, and cerebral palsy [2,3]. The complex nature of dysarthria, influenced by underlying pathology and individual patient characteristics, presents significant challenges in both assessment and management [4]. Effective assessment of dysarthria is crucial not only for understanding its severity but also for monitoring disease progression and tailoring therapeutic interventions [5].

Auditory-perceptual evaluations by speech-language pathologists remain the standard, but they are subjective and inconsistent, highlighting the need for objective and standardised tools [6]. With advancements in technology, automated, machine learning-based tools have emerged as promising alternatives, offering the potential for more objective, efficient and accessible dysarthria assessments which can be especially advantageous for individuals facing mobility challenges due to co-occurring physical disabilities [7].

Recent studies [8–13] have explored a variety of machine-learning techniques for automating the assessment of dysarthria, highlighting their potential to revolutionise diagnostics in this field. Gupta et al. [8] employed short-duration speech segments analysed via Residual Neural Networks (ResNet) to classify dysarthria severity levels. Shih et al. [9] developed an integrated model combining convolutional neural networks and gated recurrent units to detect dysarthria. Joshy et al. [10] utilised deep neural networks to classify dysarthria through low-dimensional feature representations derived from subspace modelling. Tong et al. [12] proposed a cross-modal deep learning framework that integrates both audio and video data to classify dysarthria severity levels. In a recent study, Joshy et al. [13] examined the effectiveness of multi-head attention mechanisms and multi-task learning in the automated classification of dysarthria severity levels. Lastly, Tripathi et al. [11] used DeepSpeech-based features with an SVM classifier and reported the highest accuracy of 53.9% under a leave-one-subject-out setting on UA-Speech [14], the commonly used English language data set for research of dysarthric speech.

Despite these advancements, developing accurate and reliable automated tools remains a significant challenge [15]. The variability in speech patterns among individuals with dysarthria, which is influenced by the type and severity of the underlying neurological condition, complicates the development of effective diagnostic models. Additionally, the scarcity of extensive dysarthric speech datasets, exacerbated by the difficulties in collecting prolonged speech samples from individuals with severe dysarthria, hampers the training of advanced machine learning models that require large amounts of data [16,17].

Recent advancements in deep learning, particularly transformer models, have shown potential in various speech processing tasks [18,19]. Their ability to capture contextual information across entire input sequences makes them well-suited to model the nuanced effects of dysarthria on speech [13]. However, their application to dysarthria assessment has remained limited, particularly in speaker-independent settings where generalization is critical.

To address these limitations, we propose a novel framework for dysarthria severity classification using wav2vec 2.0 [20], a state-of-the-art self-supervised transformer model pretrained on healthy speech. Our approach incorporates a speaker-agnostic training strategy that aims to improve generalizability across unseen speakers by reducing reliance on speaker-specific cues. This study makes several key contributions to the field of automated dysarthria assessment. First, we introduce the Speaker-Agnostic Latent Regularization (SALR) framework, which incorporates a multitask learning strategy combining cross-entropy and triplet margin loss to promote speaker-invariant latent representations. Second, we benchmark SALR against a fine-tuned wav2vec 2.0 model using identical inputs and architecture. Third, we evaluate the generalizability of our method on a separate dataset. Finally, we use t-SNE visualizations and speaker predictability metrics to investigate the effects of our training strategy on latent space structure and model behavior.

## Materials and methods

### Dataset

We used the Universal Access dysarthric speech corpus (UA-Speech) [14], a comprehensive and commonly used English language dataset for dysarthric speech research. The dataset comprises recordings of spoken words from 15 subjects with dysarthria and 13 age-matched healthy controls. Each participant read three blocks of 255 words, comprising 155 repeated common words (100 digits, 26 letters, 19 computer commands, and 100 common Brown Corpus words) and 100 unique uncommon words. The unique uncommon words in each block were selected from novels in Project Gutenberg to maximise phone-sequence diversity. This resulted in a total of 765 isolated words per subject, with 455 distinct words. These recordings were captured using a seven-channel microphone array and five native American English speakers transcribed the recordings. Each subject’s speech intelligibility was calculated based on the average percentage of words correctly transcribed. Subjects were categorised into four levels of dysarthric severity based on their intelligibility ratings: very low severity(76-100% intelligible), low severity (51-75% intelligible), medium severity (26-50% intelligible), and high severity (0-25% intelligible). Table 1 shows the distribution of dysarthric speakers across the four severity levels. This highlights the dataset’s inherent class imbalance, particularly in the low and medium severity groups, each comprising only two speakers. For a detailed overview of the UA-Speech dataset, readers are referred to the original publication by Kim et al. [14]. We did not apply any resampling or class-weighting strategies to balance the dataset. Instead, we used macro-averaged accuracy and F1 scores to ensure fair evaluation across all classes despite the imbalance.

**Table 1:** Distribution of speakers across dysarthria severity levels in UA-Speech.

Severity Level	Number of Speakers
Very Low	5
Low	2
Medium	2
High	6

### Finetuning the wav2vec 2.0. model

We chose to use wav2vec 2.0 [20] for our pretrained transformer over other models like Audio Spectrogram Transformer [19] and HuBERT [21] based on empirical evidence from early experimentation. The wav2vec 2.0 model was pretrained on an expansive 960-hour dataset from diverse audiobook libraries. The underlying transformer architecture of this model consists of 12 transformer blocks. Each block has a model dimension of 768, an inner feed-forward network dimension of 3072, and 8 attention heads. We did not conduct the pretraining ourselves. We utilise the publicly available facebook/wav2vec2-base pretrained model available from the 4.33.1 version of the HuggingFace library [22]. To fine-tune our model for the specialised task of classifying dysarthria severity, we added a linear classification head comprising two linear layers with a ReLU activation. The fine-tuning training was conducted with a batch size of four, using the Adam optimiser set with a learning rate of 0.0005, betas configured to (0.9,0.98), and an epsilon value of $1\times 10^{-8}$.

### Speaker-Agnostic Latent Regularisation (SALR) framework

Our initial experiments demonstrated a significant challenge with simply fine-tuning an off-the-shelf wav2vec 2.0 model: its tendency to overfit specific speakers. This could be attributed to the limited diversity within the UA-Speech dataset, which only includes 15 distinct dysarthric speakers. Instead of effectively learning the characteristics specific to dysarthria severity, the model appears to be leveraging speaker-specific cues. This approach can minimise the training loss, through recognising the speaker’s identity and subsequently assigning a dysarthria severity label. But this approach struggles with new, previously unheard speakers, highlighting a gap in the model’s ability to generalise. This issue extends to the latent space, potentially leading to the formation of speaker-centric clusters. Words from the same speaker cluster more closely than identical words from different speakers, even at the same severity. This entangled representation of words is because the complexity of a word — defined by its syllables, phonetic structures, and the necessary motor control for pronunciation — directly impacts how prominently dysarthric symptoms manifest. Without a clear representation in the latent space that accounts for word complexity, the model faces challenges.

**Fig 1:**
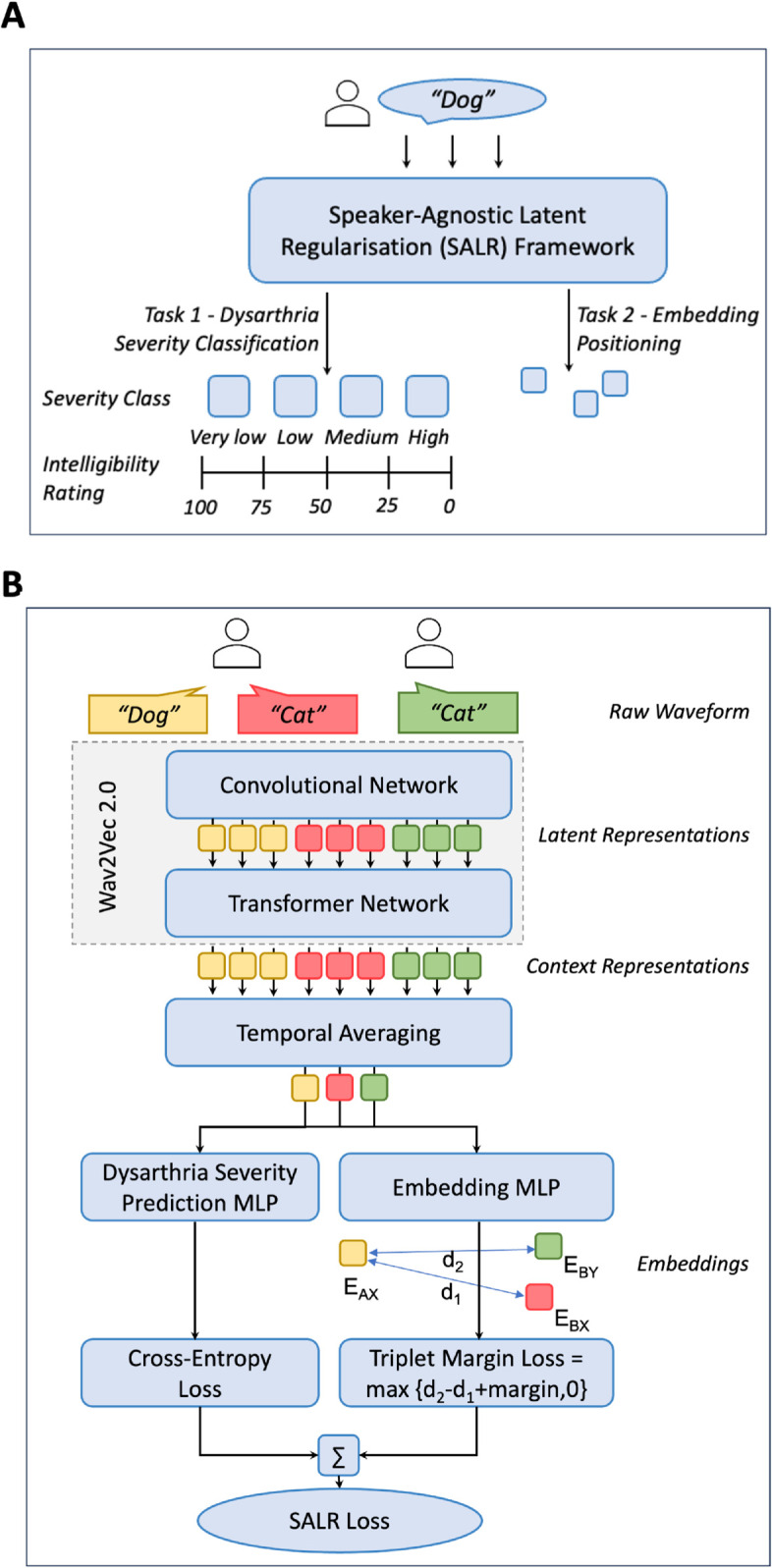
Speaker-Agnostic Latent Regularisation (SALR) framework. **A.** Conceptual overview of the SALR framework, illustrating its multi-task learning approach. The framework incorporates a primary task of dysarthria severity classification and an auxiliary task utilising contrastive learning to generate speaker-agnostic embeddings in the latent space. **B.** Detailed architecture of the SALR Framework, highlighting the computation pathways for the combined SALR loss, which includes both cross-entropy and triplet margin losses to optimise embedding separation and accuracy.

To address these issues in the latent space, we introduce a regularisation contrastive loss framework called Speaker-Agnostic Latent Regularisation (SALR) to disentangle the embeddings. Our framework (Fig 1) represents a specialised configuration that enhances the fine-tuned wav2vec 2.0 model with additional components tailored to accomplish an auxiliary task alongside the primary dysarthria classification. The auxiliary task in this framework is a contrastive learning task which aims to ensure that the separation between word embeddings within a shared severity classification becomes speaker-agnostic, thereby preventing the model from learning embeddings that embed speaker-specific characteristics. Specifically, the framework consists of an extra head designed for the auxiliary task, a weighted loss function crafted to balance the learning objectives of both the primary and auxiliary tasks, and a training regimen that specifies how the weighted loss function is applied.

To illustrate this framework, let *E*_*AX*_ denote the embedding of ‘Word A’ spoken by ‘Patient X’, *E*_*BX*_ the embedding of ‘Word B’ spoken by the same patient, and *E*_*BY*_ the embedding of ‘Word B’ spoken by a different patient with the same severity. We define the distances as $d_{1}=d(E_{AX},E_{BX})$ and $d_{2}=d(E_{AX},E_{BY})$. Our objective is to enforce $d_{1}\approx d_{2}$, so that embeddings reflect severity and word content rather than speaker identity. Initially, we hypothesise that *d*_1_ will be smaller than *d*_2_. This is because the distance *d*_1_ captures the distance between words spoken by the same speaker, and as previously discussed, our latent space tends to be influenced by speaker-specific traits.

To achieve our objective, we utilise triplet margin loss [23] with specific aims. First, we intend to push away *E*_*BX*_ from the anchor *E*_*AX*_ by considering *E*_*BX*_ as the negative sample and *E*_*AX*_ as the anchor. Given that these embeddings originate from the same speaker, we expect them to be closely located in the latent space. Thus to balance *d*_1_ and *d*_2_, the distance *d*_1_ needs to be expanded. Simultaneously, we aim to pull *E*_*BY*_ closer to *E*_*AX*_ by designating *E*_*BY*_ as the positive sample and retaining *E*_*AX*_ as the anchor. Since these embeddings are from different speakers, we hypothesise that their distance in the latent space will be larger. Thus to equate *d*_1_ and *d*_2_, the distance *d*_2_ should be contracted. Triplet margin loss ensures anchors are closer to positives than negatives by at least margin *m*. By intentionally keeping *m* minimal and taking into account the initial distances between the embeddings, we aim to make the distances between the anchor-positive and anchor-negative pairs approximately the same.

We hypothesise that implementing this regularising loss will be beneficial because it shifts the model’s focus from identifying speakers to distinguishing words. Specifically, the model should be able to differentiate between two words regardless of whether they are spoken by the same person or by different individuals with the same dysarthria severity. For example, by creating a greater distance between the anchor embedding *E*_*AX*_ and *E*_*BX*_, the model is forced to learn an embedding that focuses on the differences between the two words rather than relying on the speaker’s identity thereby making the embeddings speaker-independent given a severity class. This enhanced ability to discriminate between words allows for more accurate comparisons as it can disentangle the complexity of the word from the dysarthria.

The triplet margin loss (TML) is defined as:

$$\text{TML}(E_{AX},E_{BY},E_{BX})=\max(0,\;d(E_{AX},E_{BX})-d(E_{AX},E_{BY})+m)$$ (1)

In Eq 1, *E*_*AX*_ acts as the anchor, *E*_*BY*_ is the positive sample, and *E*_*BX*_ is the negative sample. The term *m* is a predefined margin set to 0.05. The distance function *d*(*x*,*y*) is chosen to be the *L*_2_ Euclidean distance.

The final loss *L* is expressed as $L=\epsilon L_{\text{reg}}$  +  $\gamma L_{\text{CE}}$, where ϵ serves as the weighting parameter, and is set at 0.01. The parameter γ starts at 0 for 3000 steps, allowing the model to focus on contrastive regularisation. It is then updated to 1, incorporating cross-entropy loss into the training regimen. These parameters were determined through experimentation on the 10 control patients, data we do not use in training or testing. This helps to show the robustness and generalisability of our approach, as the parameters were not tuned on data from the control group, thereby validating its potential for real-world clinical applications. To validate training stability, we plotted the training and validation loss curves across epochs for the SALR model (S1 Fig). These curves indicate stable convergence and no significant overfitting, supporting the robustness of our training regimen.

### Comparative models for evaluation

To contextualise our findings, we first compare our results against three historical models drawn from previous literature: XGBoost [24], Multi-layer Perceptron (MLP) [25], and CNN-LSTM [26]. These models differ in both architecture and input representation. XGBoost is a gradient-boosted decision tree algorithm known for its speed and performance in classification tasks [24]. MLP is a class of feedforward artificial neural networks composed of fully connected layers and nonlinear activation functions [25]. XGBoost and MLP operate on engineered acoustic features derived from the extended Geneva Minimalistic Acoustic Parameter Set (eGeMAPS) [27], an extensive set of 88 acoustic features for speech analysis. CNN-LSTM is a hybrid neural network architecture combining convolutional layers to extract spatial features and LSTM layers to capture temporal dependencies in sequential data [26]. The CNN-LSTM model processes spectrogram inputs in an end-to-end manner. To optimise the hyperparameters of the historical comparator models, we employed Bayesian optimisation using the Optuna library [28]. This method systematically explores the hyperparameter space to find the optimal set, striking a balance between exploration and exploitation.

While these comparisons provide historical context, they are not our principal comparators for evaluation due to differences in input modality. Instead, our primary comparator is the fine-tuned wav2vec 2.0 model, which uses the same raw waveform inputs and transformer architecture as our proposed Speaker-Agnostic Latent Regularisation (SALR) framework. This controlled comparison ensures that any improvements in performance can be directly attributed to the introduction of our multi-task contrastive learning objective, rather than architectural or input differences.

### Ethics statement

The UA speech dataset employed in this study was previously published [14] and is accessible upon request, subject to ethical considerations by the original authors. The TORGO dataset [4] is publicly available [29]. All participants in the datasets were adults (over 18 years of age) and provided explicit consent for the use and dissemination of their data. Our use of the dataset aligns with the original consent and required no further approval from our Institutional Review Board.

## Results

### Speaker-dependent vs speaker-independent splits

In our investigation, we examined both speaker-dependent and speaker-independent data splits for training and evaluating models for dysarthric speech severity classification. The speaker-dependent split facilitates the model’s training and testing on data from the same individuals, albeit with different words during the testing phase. Although this method aids in model training due to the consistency of voice patterns, its applicability in a clinical environment is restricted, as it fails to validate the model against new patients—a fundamental requirement for an automated diagnostic tool. Consequently, we focused on the speaker-independent data split setup, in which the model is trained and assessed on data from distinct groups of speakers. This ensures the model’s capacity to generalise across unfamiliar voices. Our study presents findings on the speaker-independent multi-class severity classification task, requiring the model to categorise the severity of dysarthric speech into four distinct levels: very low, low, medium, and high. This approach is vital as it aligns directly with clinical relevance and the model’s efficacy across diverse patient conditions.

### Speaker-independent multi-class severity results

In this study, we evaluated model performance using a rigorous leave-one-subject-out cross-validation method. With a total of 15 speakers in our dataset, we conducted 15 iterations of training and testing, recording the average test results. In each iteration, data from 14 subjects (comprising 465 utterances for each subject, with three repetitions of 155 digits/alphabets/common words from the dataset) were used for training. The 300 uncommon words from the remaining subject were used for testing. This process was systematically repeated for all 15 subjects, ensuring each subject’s data was exclusively utilised for testing once. This rigorous methodology guarantees the robustness and reliability of our findings for not only new dysarthric patients but also new vocabulary. The leave-one-subject-out cross-validation process was repeated five times, with mean and standard deviation values recorded.

To ensure fairness in evaluation, we used a fine-tuned wav2vec 2.0 model trained solely with cross-entropy loss on raw waveform inputs as our primary comparator. This model shares the same architecture and input modality as our proposed SALR framework, providing a direct and rigorous benchmark. The fine-tuned wav2vec 2.0 model achieved an accuracy of 64.81±1.26%. The SALR framework, which introduces a triplet margin loss for contrastive regularisation, achieved a significantly higher accuracy of 70.48±1.11% and an F1 score of 59.23±1.54%. This 16.5% absolute improvement (or over 30% relative) demonstrates the benefit of our multi-task learning objective.

Tables 2 and 3 also report results from historical comparators—XGBoost, MLP, and LSTM-CNN—drawn from prior work. These models operate on different input representations (engineered features or spectrograms) and achieved accuracies below 50%. While informative for context, these historical models are not directly comparable due to their differing input modalities.

**Table 2:** Table comparing the performance of various models, presenting mean ± standard deviation of accuracy scores for each of the 15 patients across five iterations of leave-one-subject-out cross-validation.

Patient Code	MLP	LSTM-CNN	XGBoost	Finetuned wav2vec 2.0	SALR
M04	51.78 ± 1.84	73.49 ± 2.34	62.83 ± 2.98	87.19 ± 0.95	79.62 ± 0.79
F03	57.39 ± 3.22	70.32 ± 4.33	61.91 ± 2.43	89.46 ± 0.84	77.00 ± 0.67
M12	61.89 ± 1.84	74.47 ± 2.33	71.89 ± 2.42	92.40 ± 0.74	88.64 ± 0.93
M01	56.74 ± 2.33	60.83 ± 3.42	63.84 ± 1.89	78.13 ± 1.09	81.18 ± 0.93
M07	15.98 ± 5.84	7.38 ± 4.84	10.18 ± 4.33	7.67 ± 1.89	20.00 ± 1.57
F02	9.39 ± 3.84	5.38 ± 3.33	7.85 ± 5.75	22.74 ± 1.89	21.20 ± 2.39
M16	13.73 ± 4.39	8.48 ± 4.72	11.43 ± 3.27	26.02 ± 2.00	19.12 ± 1.32
M11	8.48 ± 3.28	13.49 ± 5.47	13.43 ± 4.33	32.49 ± 1.04	58.10 ± 1.32
F04	6.48 ± 3.82	7.43 ± 4.37	11.85 ± 4.46	25.83 ± 2.38	61.60 ± 1.01
M05	9.04 ± 4.84	9.49 ± 5.79	16.89 ± 4.23	26.58 ± 1.00	60.53 ± 1.39
M09	73.38 ± 2.38	72.78 ± 2.80	81.89 ± 2.89	92.58 ± 0.89	98.43 ± 0.89
M08	72.37 ± 2.80	80.41 ± 3.24	78.94 ± 1.98	95.55 ± 0.79	97.20 ± 0.71
M10	76.47 ± 1.98	75.49 ± 1.32	81.89 ± 2.89	96.05 ± 1.84	97.70 ± 0.90
M14	77.90 ± 2.81	79.95 ± 1.39	79.80 ± 1.39	97.98 ± 0.67	98.10 ± 0.90
F05	74.24 ± 3.81	72.38 ± 1.43	80.84 ± 1.23	95.36 ± 0.84	98.80 ± 0.93
Average	44.35 ± 3.27	47.45 ± 3.34	49.03 ± 2.98	64.81 ± 1.26	**70.48** ± 1.11

**Table 3:** Table comparing the performance of various models, presenting mean ± standard deviation of F1 score across five runs of leave-one-subject-out cross-validation.

MLP	LSTM-CNN	XGBoost	Finetuned wav2vec 2.0	SALR
27.44 ± 3.83	29.95 ± 4.15	37.75 ± 3.20	52.39 ± 2.13	**59.23** ± 1.54

**Fig 2:**
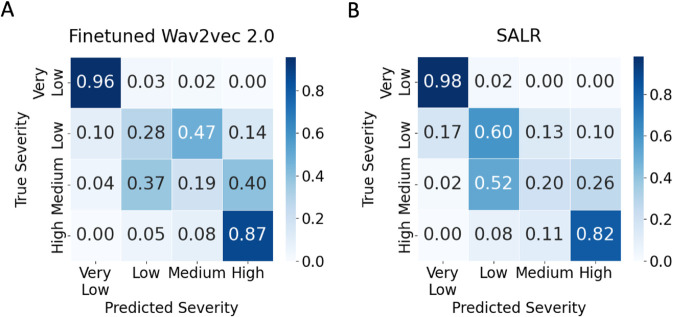
Normalised confusion matrices. **A.** Fine-tuned wav2vec 2.0 model **B.** SALR framework

While aggregate metrics such as F1 score and accuracy provide substantial insights, further insight is obtained through the analysis of the confusion matrices. The confusion matrices (Fig 2) illustrate our models’ proficiency in classifying extreme dysarthric severities but also highlight challenges in differentiating between low and medium severity classes. Specifically, the fine-tuned wav2vec 2.0 model (Fig 2A) frequently misclassified instances of low as medium severity and medium as low and high. In comparison, the SALR framework (Fig 2B) struggled with distinguishing medium instances, often mis-classifying medium as low severity.

**Fig 3:**
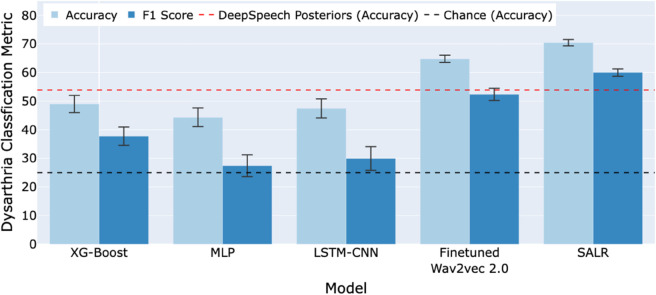
Comparative performance of various models for speaker-independent multi-class dysarthria severity classification on the UA-Speech dataset. Accuracy and F1 scores of our models compared to chance predictions and the existing benchmark set by Tripathi et al. [11] using DeepSpeech posteriors. Error bars represent the standard deviation across five repetitions, illustrating the consistency of model performance.

To contextualise our findings within the broader scope of existing research, we compared our results with those from previous studies on speaker-independent dysarthria classification. Tripathi et al. [11] reported the highest accuracy of 53.90% using features obtained from DeepSpeech—a deep learning-based speech-to-text engine—combined with an SVM classifier under a leave-one-subject-out cross-validation scheme. In contrast, our initial results using a fine-tuned wav2vec 2.0 model showed a better classification accuracy of 64.81%. We achieved further improvements using our SALR framework, which reached an accuracy of 70.48% (see Fig 3). This comparative analysis highlights the significant advancements made by our SALR framework over previous methods. Utilising the same test set and cross-validation scheme, our study ensures a rigorous and fair comparison, demonstrating notable enhancements in methodological approach and classification accuracy, crucial for effective implementation in various clinical settings.

Comparative performance of various models for speaker-independent dysarthria severity classification, tested for new speakers but not new vocabulary [by testing on all words of the test subject], is presented in S2 Fig and S3 Fig.

To assess generalizability, we evaluated SALR on a new TORGO dataset [4] using the same leave-one-subject-out cross-validation. As summarised in Table 4, SALR outperformed both traditional models and the fine-tuned wav2vec 2.0 comparator, suggesting SALR’s robustness across datasets despite differences in speakers and recording conditions.

**Table 4:** Performance metrics (mean ± SD) for dysarthria severity classification on the TORGO dataset across five runs of leave-one-subject-out cross-validation.

Model	F1 Score	Accuracy
MLP	21.19 ± 4.59	39.42 ± 2.40
LSTM-CNN	24.13 ± 3.98	42.78 ± 4.75
XGBoost	33.61 ± 4.34	41.38 ± 3.62
Finetuned wav2vec 2.0	46.12 ± 3.70	59.27 ± 3.90
SALR	**51.79** ± 2.26	**64.45** ± 2.39

### Speaker predictability

To quantify the degree of speaker-specific information present in the model embeddings, we trained a linear classifier on the latent representations to predict speaker identity on the UA-Speech dataset. As shown in Table 5, embeddings from the fine-tuned wav2vec 2.0 model yielded a speaker predictability of 70.28% ± 4.85, whereas the SALR embeddings resulted in a significantly lower accuracy of 37.53% ± 5.48. This reduction indicates that the SALR framework effectively suppresses speaker-specific cues, promoting more speaker-invariant representations.

**Table 5:** Accuracy scores (mean ± SD) for speaker predictability on the UA-Speech dataset using a linear classifier on embeddings, across five runs of leave-one-subject-out cross-validation.

Model	Accuracy
Finetuned wav2vec 2.0	70.28 ± 4.85
SALR	**37.53** ± 5.48

### Interpretation of the latent space analysis

**Fig 4:**
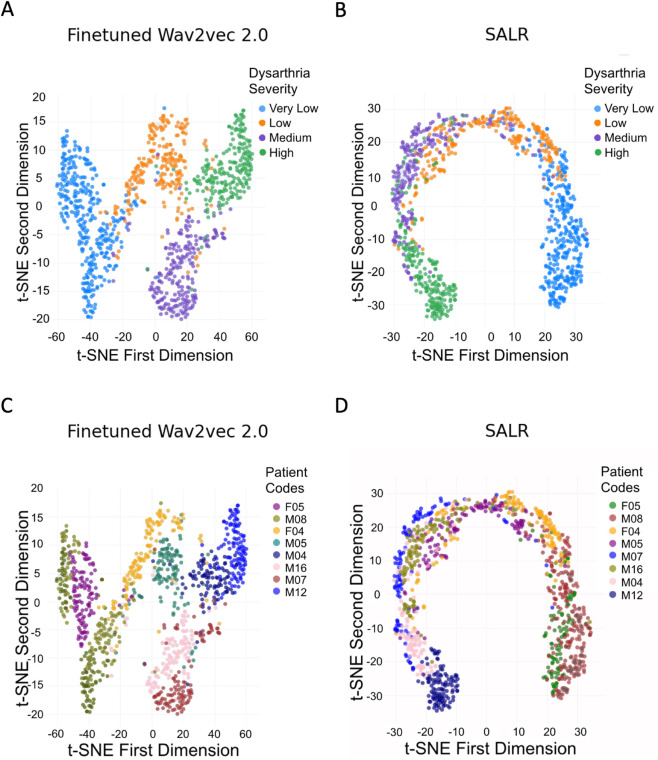
Visualisation of t-SNE embeddings. Data points are coloured according to patient severity (first row) and patient code (second row) for the fine-tuned wav2vec 2.0 model **(A, C)** and the SALR framework **(B, D)**. These visualisations support our hypothesis that the SALR framework organises the latent space in alignment with severity levels (first row) and disperses speaker clusters ( second row).

To further assess the impact of our frameworks on the model’s representation of speech data, we conducted a t-SNE analysis of the latent space. Fig 4 provides visual insights into how the models organise the latent representations of both the fine-tuned wav2vec 2.0 model and the SALR framework with respect to dysarthria severity and speaker identity. For clarity, the t-SNE plots display a randomly selected subset of eight speakers (two from each severity class) to maintain class representation while avoiding overcrowding and ensuring that overall trends remain visible.

In the fine-tuned wav2vec 2.0 model, the latent space displays a lack of structured organisation with respect to ordinal severity levels. High-severity samples often cluster closely to both mid and low-severity samples (Fig 4A). Additionally, distinct clusters corresponding to different speakers are evident (Fig 4C). In contrast, the SALR multi-task framework introduces a clearer ordinal structure to the latent space (Fig 4B). Speaker clusters within this framework are also more dispersed (Fig 4D). These observations highlight the effectiveness of the contrastive loss in our SALR framework, which successfully disentangles speaker-specific cues from severity assessments in the latent space.

To complement the qualitative t-SNE visualisation, we quantitatively assessed speaker clustering using a linear classifier trained to predict speaker identity from the model embeddings (Sect 3.3). The classifier achieved significantly lower accuracy on embeddings from the SALR model compared to the primary comparator model, the fine-tuned wav2vec 2.0, confirming that SALR reduces speaker-specific information in the latent space. While some residual clustering remains, these results support our hypothesis that reducing speaker leakage improves generalisability in speaker-independent dysarthria severity classification. Our findings align with prior work on domain-adversarial training [30], which demonstrates that reducing domain-specific information can enhance generalisation.

## Discussion

The primary focus of the study was developing an automated, machine learning-based tool for classifying dysarthria severity levels in a speaker-independent manner. We first fine-tuned a base wav2vec 2.0 model, a state-of-the-art self-supervised transformer model, trained on healthy speech for the task of dysarthria severity assessment. The fine-tuned model outperformed other traditional models based on XGBoost, MLP, and LSTM-CNN. Our analysis indicated that the model could achieve more accurate results by focusing on dysarthria-specific speech features rather than on individual idiosyncrasies unrelated to the actual severity of the condition. To counteract the model’s tendency to overfit to individual speakers and improve generalisation, we introduced the novel SALR multi-task framework. This framework significantly improved the model’s performance, achieving an accuracy of 70.48% and an F1 score of 59.23%, marking a 16.58% increase over the published benchmark. Further analysis shows that the SALR multi-task objectives not only enhance numerical performance but also help organise the latent space in a manner that aligns with severity levels. This reduces the model’s reliance on speaker-specific cues, thus boosting performance and validating our hypothesis about the effects of the multi-task framework.

Using confusion matrices for speaker-independent evaluation, we found that while the framework excels in categorising extreme severity classes, it faces challenges in distinguishing between low and medium severity levels. These challenges are primarily attributable to the limited number of patient samples available for these categories post-segmentation, with only two patients remaining in each of the low and medium categories, thereby limiting the model’s learning efficacy. Compounding this issue is the lack of distinct boundaries between these classes. For example, patient M16, classified as medium severity with 43% intelligibility, is much closer to the low category (M05: 58%) than to others in the medium class (M07: 28%, F02: 29%). Consequently, the model’s ability to accurately classify ambiguous cases like M16 may be compromised due to the combined factors of data sparsity and ambiguous class distinctions.

While the use of transformer-based frameworks is effective, it introduces challenges related to interpretability. Although our latent space visualisation provides some insights into the model’s functionality, additional methods, such as attention heatmaps [31], layer-wise relevance propagation, or saliency maps [32], could highlight key acoustic features driving severity predictions. This is particularly important as we move toward automated dysarthria severity assessments, where transparency and interpretability are crucial.

While our approach demonstrates strong performance and introduces novel methodological advances, these findings should be interpreted in light of several limitations. Generalisation is limited by the small, imbalanced UA-Speech dataset and its focus on American English. Validation on TORGO shows robustness, but broader testing across languages and accents is needed. The model’s 70.5% accuracy in the UA-Speech dataset, although improved over benchmarks, is insufficient for standalone clinical use, and its performance has not been compared to human raters. Deployment in real-world contexts will require prospective validation, noise-robustness testing, and clinician-in-the-loop systems to guarantee equitable and transparent outcomes [33,34]. Using wav2vec 2.0 pretrained on healthy speech may introduce representational bias, as the model is not exposed to atypical patterns in dysarthric speech during pretraining. It may limit the model’s ability to fully capture disorder-specific features. Future work could address this through domain adaptation [30] or pretraining on dysarthric speech corpora. Furthermore, while intelligibility percentage is widely used in existing datasets like UA-Speech and TORGO, it captures only one dimension of dysarthria severity. It may miss important clinical features such as prosody, articulation, and voice quality. Future research should explore multi-dimensional labels (e.g., perceptual ratings or dysarthria subtypes) to enable more clinically valid modelling.

Addressing these limitations will be key to advancing SALR toward clinical deployment. Future work should validate the framework on larger and more diverse datasets, including multiple languages, accents, and varied recording conditions, and benchmark its performance against expert human raters. Comparative studies are also needed to examine the relative benefits of speaker-independent models versus personalised baseline approaches, which may better reflect the inherently individualised nature of clinical dysarthria assessments. Additionally, comparisons with other transformer-based models such as HuBERT [21], trained on raw waveform inputs but using different self-supervised learning objectives, will provide a more rigorous and architecture-consistent benchmark for evaluating SALR. Future work could also explore benchmarking wav2vec 2.0 embeddings across multiple classifier architectures (e.g., XGBoost, MLP, or CNN-based models). Such analyses would help disentangle the contribution of pretrained feature representations from model-specific training objectives and further validate the robustness of SALR. While SALR promotes speaker-agnostic representations, its performance on underrepresented or minority groups remains untested. Broader validation across these populations is essential for equitable deployment. Future work should also consider moving beyond intelligibility-based labels, which may not capture the full clinical complexity of dysarthria. Exploring multi-dimensional annotations or unsupervised learning could help uncover richer severity representations grounded in articulation, prosody, and voice quality. SALR is flexible and can be adapted to richer clinical targets, such as perceptual ratings, multi-domain scales, or applied to non-English datasets through multilingual transformer models like wav2vec 2.0. In future work, SALR could be combined with domain adaptation [30] or meta-learning strategies to further mitigate overfitting and improve generalisation to previously unseen speakers and clinical populations. While our t-SNE visualisations provide qualitative insight, future work should include quantitative evaluation of the high-dimensional embeddings. Metrics such as intra- vs. inter-class distance ratios or linear separability scores [35,36] can provide a more rigorous assessment of alignment with clinical severity labels, independent of projection artefacts from t-SNE. Finally, incorporating explainability techniques such as attention heatmaps [31] and relevance propagation [37], alongside fairness audits to assess potential demographic or socio-linguistic biases, will be critical to ensure AI equity and ethical deployment across diverse clinical populations. While our t-SNE visualisations suggest improved alignment with severity levels, future work should include quantitative metrics (e.g., cluster separability or embedding distance analysis) to more rigorously evaluate how well the latent space captures clinical severity distinctions.

We also acknowledge the ethical implications of deploying AI-based dysarthria assessment tools in clinical settings. Our model is not intended to replace human raters, but rather to support clinician-in-the-loop decision-making workflows [33]. Any future deployment would require not only rigorous clinical validation but also safeguards to ensure transparency, explainability, and fairness across diverse populations. Ethical deployment must also consider patient consent, data privacy, and the need for clear accountability mechanisms in the diagnostic process [34].

Our findings demonstrate technical promise that, with further validation, could inform future clinical applications in dysarthria assessment. The ability of the SALR framework to provide reliable and accurate assessments of dysarthria severity in a speaker-independent manner is particularly relevant for clinical settings. Integrating automated tools like our framework in clinical practice could significantly enhance diagnostic processes. If validated in larger and more diverse datasets, this advancement could facilitate more objective and efficient assessments of dysarthria, contributing to improved patient care and management [38]. SALR offers the opportunity to reduce the unmet demand for speech and language assessments in terms of both quantity and quality. This is particularly important for healthcare systems strained by staff shortages, rapidly ageing populations, and increased healthcare service demand. Speech and language therapy is a profession with substantial training requirements, limiting the availability of experts in many countries. For example, the UK has a vacancy rate of around 25% and recognises it as a shortage profession [39]. AI-based speech assessment could support diagnostic assessments and assist in training professionals, particularly in the initial stages of their training, e.g. see [40,41]. In daily operations, this technology could be used in conjunction with human raters or as an autonomous system for rapid initial assessments; or, with more training data, for systematic assessments.

Although wav2vec 2.0 pretraining is resource-intensive [20], SALR uses public pretrained weights [22] and requires only modest resources for fine-tuning, making it practical for small clinical datasets. The accessibility and cost-effectiveness of our AI-based approach could enhance the speed and precision of dysarthria assessments, particularly benefiting individuals with mobility challenges due to co-occurring physical disabilities [42] and thus potentially allow assessment via videoconferencing, as in dermatology [43]. Crucially, remote or home assessment would enable true patient-centric evaluation of patient capability, a rapidly growing domain of digital healthcare [44,45]. Thus, speech rehabilitation of dysarthria could be potentially even entirely technologically guided as in other forms of motor rehabilitation in a multi-modal, multi-sensory AI-guided treatment in the real world [46,47] for smart rehabilitation. However, any real-world deployment would require careful assessment and comparison of commercial and clinical-grade speech recording methodologies, as was done in other domains of sensing [48].

## Conclusion

This proof-of-concept study demonstrates that the Speaker-Agnostic Latent Regularisation framework achieves markedly improved classification performance compared to existing benchmarks and advances speaker-independent dysarthria severity classification by reducing reliance on speaker-specific cues and improving the organisation of latent representations. Broader validation across larger, multilingual, and clinically diverse datasets, along with direct comparison to expert raters, will be essential to determine clinical applicability. Our findings provide preliminary evidence that contrastive multi-task learning can overcome key limitations of existing models, underscoring the potential of this approach to support the development of more objective and scalable tools for dysarthria assessment.

## Supporting information

S1 Fig Training and validation loss plots for SALR for a single split of the leave-one-subject-out cross-validation on the UA-Speech dataset, showing stable convergence. (TIF)

S2 Fig Comparative performance of various models for speaker-independent multi-class dysarthria severity classification on the UA-Speech dataset (tested on all words of the test subject using leave-one-subject-out cross-validation). We have plotted the accuracy and F1 scores of our models compared to chance predictions for the case when all 765 utterances from the test subject were used in the test set. This test case checks for system performance for new speakers (but not new vocabulary). Error bars represent the standard deviation across five repetitions.
(TIF)

S3 Fig Normalised confusion matrices for the case when all 765 utterances from the test subject were used in the test set. **A.** fine-tuned wav2vec 2.0 model, **B.** SALR framework.
(TIF)
