# Supplementary figures and images for: Speaker-independent dysarthria severity classification using self-supervised transformers and multi-task learning
Source: PLOS Digit Health. 2025 Nov 12;4(11):e0001076. doi: 10.1371/journal.pdig.0001076 (PMC12611135; doi:10.1371/journal.pdig.0001076)

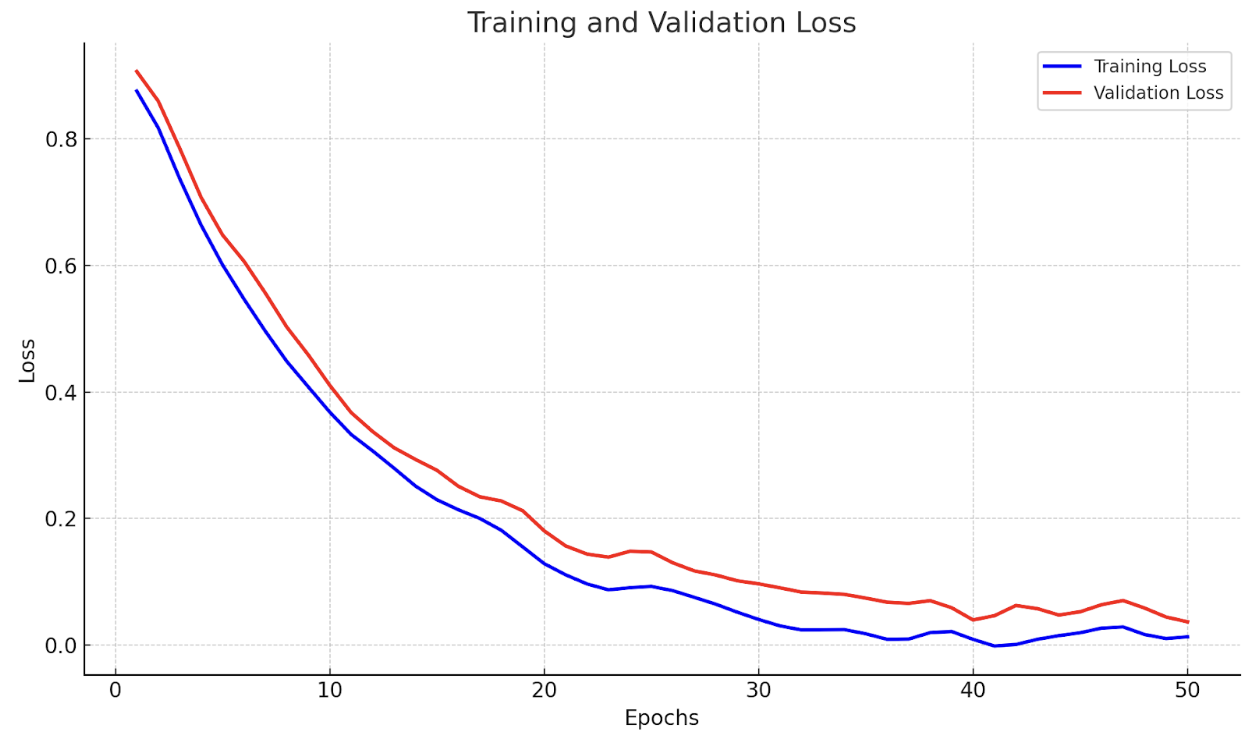

Supplement: S1 Fig — (TIF) [file pdig.0001076.s001.tif]

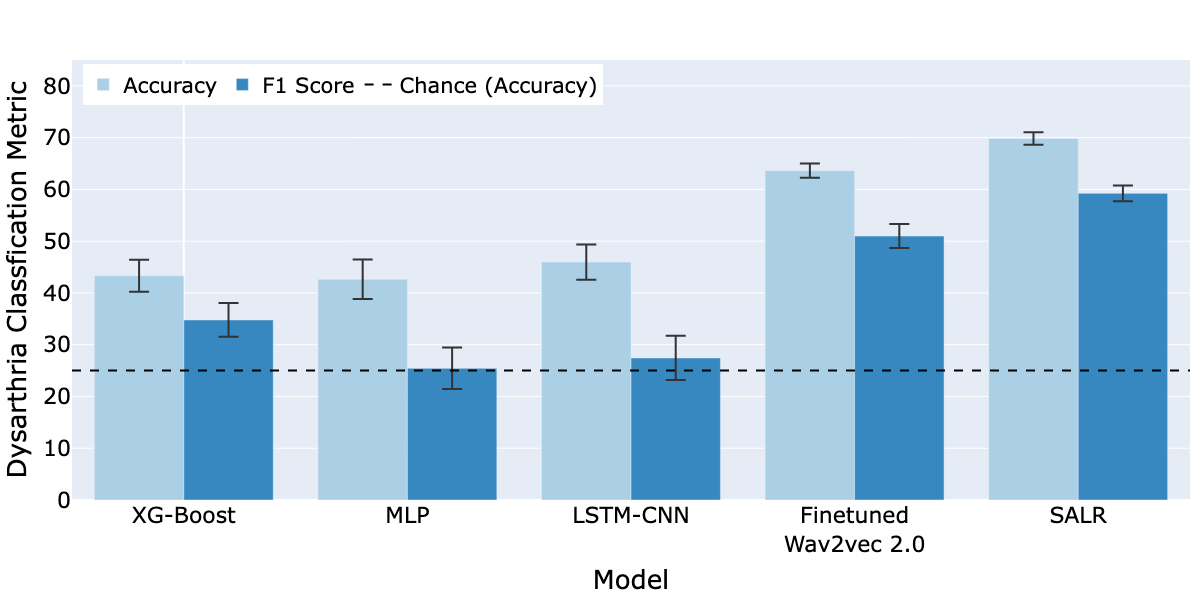

Supplement: S2 Fig — We have plotted the accuracy and F1 scores of our models compared to chance predictions for the case when all 765 utterances from the test subject were used in the test set. This test case checks for system performance for new speakers (but not new vocabulary). Error bars represent the standard deviation across five repetitions. (TIF) [file pdig.0001076.s002.tif]

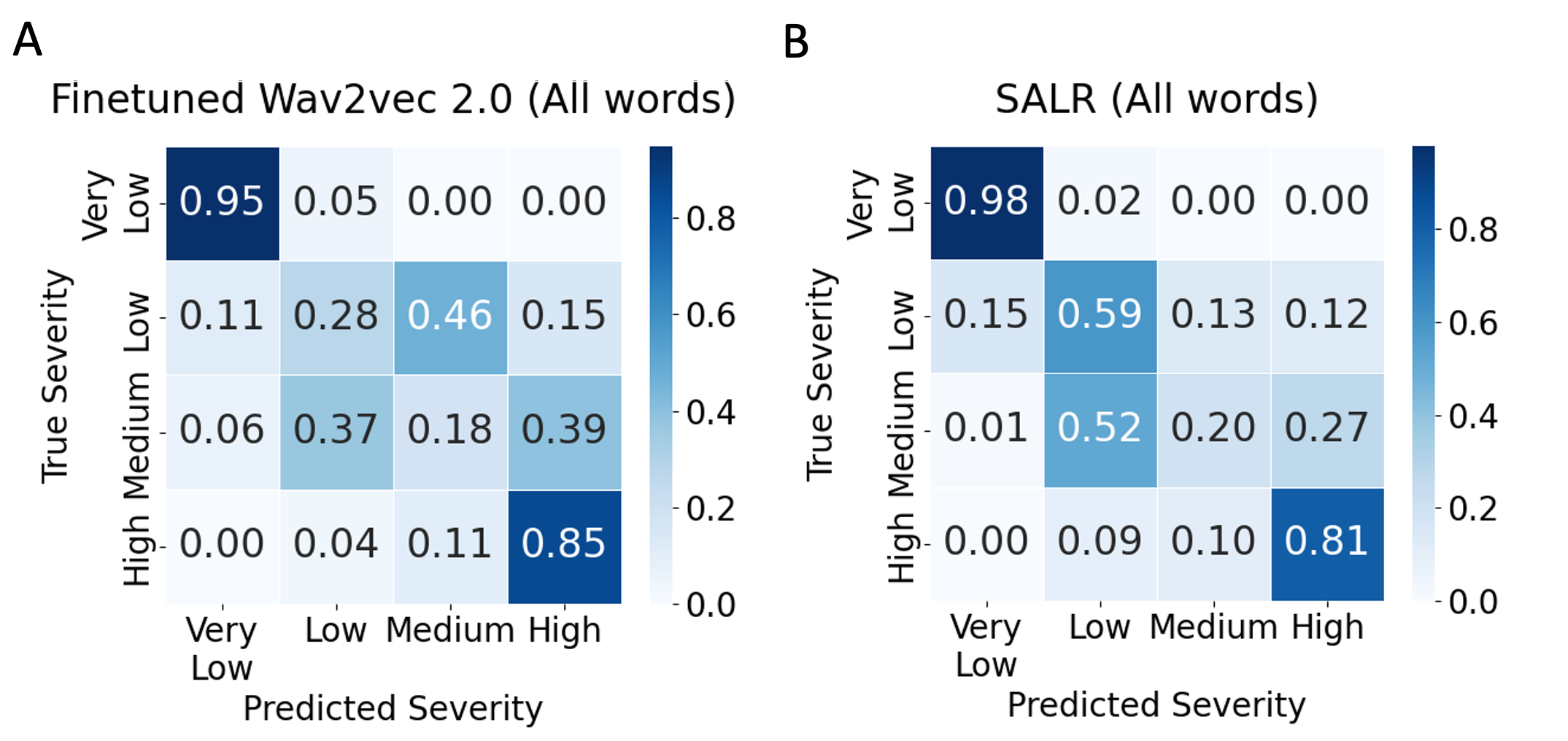

Supplement: S3 Fig — A. fine-tuned wav2vec 2.0 model, B. SALR framework. (TIF) [file pdig.0001076.s003.tif]
